# Pro-inflammatory cytokines and bone fractures in CKD patients. An exploratory single centre study

**DOI:** 10.1186/1471-2369-13-134

**Published:** 2012-10-09

**Authors:** Vincenzo Panuccio, Giuseppe Enia, Rocco Tripepi, Roberta Aliotta, Francesca Mallamaci, Giovanni Tripepi, Carmine Zoccali

**Affiliations:** 1 Nephrology, Dialysis, Hypertension and Renal Transplantation Unit Azienda Ospedaliera, Via vallone Petrara, 89124, Reggio Calabria, Italy; 2 CNR-IBIM Clinical Epidemiology and Physiopathology of Renal Diseases and Hypertension, Via vallone Petrara, 89124, Reggio Calabria, Italy

**Keywords:** Bone fractures, CKD, Dialysis, Hyperparathyroidism, TNF-alpha, Inflammation

## Abstract

**Background:**

Pro-inflammatory cytokines play a key role in bone remodeling. Inflammation is highly prevalent in CKD-5D patients, but the relationship between pro-inflammatory cytokines and fractures in CKD-5D patients is unclear. We studied the relationship between inflammatory cytokines and incident bone fractures in a cohort of CKD-5D patients.

**Methods:**

In 100 CKD-5D patients (66 on HD, 34 on CAPD; males:63, females:37; mean age: 61 ± 15; median dialysis vintage: 43 months) belonging to a single renal Unit, we measured at enrolment bone metabolic parameters (intact PTH, bone and total alkaline phosphatase, calcium, phosphate) and inflammatory cytokines (TNF-α, IL-6, CRP). Patients were followed-up until the first non traumatic fracture.

**Results:**

During follow-up (median: 74 months; range 0.5 -84.0) 18 patients experienced fractures. On categorical analysis these patients compared to those without fractures had significantly higher intact PTH (median: 319 pg/ml IQ range: 95–741 vs 135 pg/ml IQ: 53–346; p = 0.04) and TNF-α levels (median: 12 pg/ml IQ: 6.4-13.4 vs 7.8 pg/ml IQ: 4.6-11; p = 0.02). Both TNF-α (HR for 5 pg/ml increase in TNF-α: 1.62 95% CI: 1.05-2.50; p = 0.03) and intact PTH (HR for 100 pg/ml increase in PTH: 1.15 95% CI: 1.04-1.27; p = 0.005) predicted bone fractures on univariate Cox’s regression analysis. In restricted (bivariate) models adjusting for previous fractures, age, sex and other risk factors both PTH and TNF-α maintained an independent association with incident fractures.

**Conclusions:**

In our bivariate analyses TNF-α was significantly associated with incident fractures. Analyses in larger cohorts and with adequate number of events are needed to firmly establish the TNF α -fracture link emerged in the present study.

## Background

Bone mineral disorders are pervasive in patients with kidney failure on dialysis (CKD stage 5D) and the risk for bone fractures is quadrupled in this population [1,2]. Deranged parathyroid function is currently considered as the fundamental alteration responsible for bone disease in CKD [3]. Past exposure to steroids applied to treat immunological renal diseases or administered in previous kidney transplants represents an additional major factor in the pathogenesis of bone fractures in these patients [1]. Apart from parathyroid hormone (PTH) and other major hormonal regulators of bone metabolism, during the last two decades pro-inflammatory cytokines have fully emerged as major players in bone remodeling [4]. In particular, Tumor Necrosis Factor Alpha (TNF-α) a cytokine endowed with a large repertoire of biological effects is one of the most powerful inducers of the receptor activator of NF-kB ligand (RANKL), i.e. a key trigger of osteoclast activation and bone resorption [4-7]. High cytokines levels may contribute to increase the risk of osteoporosis and bone fractures in chronic inflammatory disease including COPD [8] and inflammatory bowel disease [9], and the relevance of RANKL pathway in bone health is indicated by the efficacy of drugs impinging upon RANKL in the treatment of osteoporosis in elderly women [10], including patients in CKD stage 2–4 [11]. Inflammation is a feature of advanced CKD [12-15], but the relationship between pro-inflammatory cytokines and fractures in CKD-5D patients is still unclear. To explore the hypothesis that inflammation may contribute to the high risk of bone fracture in CKD we tested the relationship between inflammatory makers and other bone metabolic parameters with incident bone fractures in a cohort of stable CKD-5D patients without inter-current clinical infectious processes.

## Methods

### Study population

The study protocol was approved by the Ethics Committee of the Azienda Ospedaliera “Bianchi-Melacrino-Morelli” di Reggio Calabria. All patients provided informed consent.

All prevalent patients in January 1995 and incident patients in 1996–1997 [66 on haemodialysis (HD) and 34 on continuous ambulatory peritoneal dialysis (CAPD), 63 males and 37 females] belonging to a single renal Unit, who had been on regular dialysis treatment (RDT) for at least 6 months and without inter-current clinical problems requiring hospitalization were recruited for the study. Patients mean age was 61 ± 15 years and the median duration of dialysis treatment was 43 months (inter-quartile range 18–99 months). Further clinical details about the study population are given in Table 1. Hemodialysis patients were being treated thrice weekly with standard bicarbonate dialysis (Na 138, HCO_3_ 35, K1.5, Ca 1.25, Mg 0.75 mmol/L) and 1.1-1.7 m^2^ dialysers (89% cuprophan, 11% semi-synthetic membranes). The average fractional urea clearance (Kt/V) in these patients was 1.28 ± 0.31. Dialysis fluid was produced by a reverse osmosis system and Aluminium never exceeded 5 μg/L which is well below the safety limit recommended by the European Council. Patients on CAPD were all on 4 exchanges/day schedule with standard dialysis bags containing 1.75 mmol/L calcium. The average weekly Kt/V in these patients was 1.67 ± 0.30. Sixteen patients were diabetics and 48 were habitual smokers. Forty-nine patients were on treatment with erythropoietin and 60 were taking various anti-hypertensive drugs (42 on mono-therapy with ACE inhibitors, calcium channel blockers or beta blockers and the remaining 18 on double or triple therapy with various combinations of these drugs). Eighteen patients out of 60 on anti-hypertensive therapy were on treatment with beta blockers (alone or in combination with other drugs). Eighty-six patients were assuming calcium chelating agents (either calcium carbonate or calcium acetate). Forty-seven patients were being treated with calcitriol. None of patients who took part in the study had undergone parathyroidectomy.

**Table 1:** Main demographic, clinical and biochemical data of patients

	**With Fractures (n.18)**	**Without Fractures (n.82)**	**p**
Age (years)	61 ± 13	61 ± 15	0.91
Male Sex (%)	61	63	0.86
Duration of RDT (months)	35 (14–75)	44 (18–101)	0.47
BMI (Kg/m^2^)	26.8 ± 4.8	25.1 ± 4.4	0.18
Height (cm)	159 ± 10	161 ± 10	0.48
Smokers (%)	50	48	0.85
Diabetics (%)	17	16	0.93
Treated with Beta blockers (%)	6	21	0.13
Treated with Calcitriol (%)	39	49	0.45
Treated with Ca-carbonate or Ca- acetate (%)	83	87	0.72
Treated with ESAs (%)	39	51	0.34
Hemodialysis/CAPD (n.)	15/3	51/31	0.09
History of previous fractures (%)	24	4	**0.009**
History of renal transplantation (%)	6	10	0.56
Haemoglobin (g/dL)	9.8 ± 2.6	10.5 ± 1.8	0.27
Serum Albumin (g/dl)	4.0 ± 0.7	3.8 ± 0.6	0.27
Serum Calcium (mmol/L)	4.5 ± 0.6	4.5 ± 0.6	0.73
Serum Phosphate (mg/dl)	6.0 ± 1.4	6.0 ± 1.6	0.92
Intact PTH (pg/mL)	319 (95–741)	135 (53–346)	**0.04**
intact PTH < 100 pg/ml (%)	28	46	0.15
intact PTH > 800 pg/ml (%)	22	6	**0.03**
Bone Alkaline phosphatase (μg/L)	15.0 (6.4-21.2)	12.7 (7.9-22.9)	0.89
Total Alkaline phospatase (UI/L)	67 (58–98)	69 (51–86)	0.88
IL-6 (pg/mL)	6.1 (3.2-8.0)	7.2 (3.3-11.0)	0.50
CRP (mg/L)	11.1 (3.5-28.2)	8.9 (3.4-19.1)	0.59
TNF-α (pg/mL)	12.0 (6.4-13.4)	7.8 (4.6-11.0)	**0.02**

Data are expressed as mean ± SD, median and inter-quartile range or as percent frequency, as appropriate. Patients are divided into 2 groups on the basis incident fractures occurrence. P tests the differences among the groups. Significant differences between groups are indicated in bold. Intact PTH thresholds of < 100 and >800 pg/ml identify low and high bone turn-over, respectively [18]. ESAs = erythropoiesis-stimulating-agents.

### Laboratory methods

Fasting blood sampling was performed during a midweek non-dialysis day for HD patients. Samples were stored in prechilled vacutainers containing edetic acid, placed immediately on ice, and centrifuged within 30 min at 4 °C; plasma was stored at −80 °C until required. Serum calcium, serum phosphate, haemoglobin and alkaline phosphatase measurements were made using standard methods in the routine clinical laboratory. Intact PTH molecule measurement was made by a specific immuno-radiometric assay (Scantibody Laboratory Inc, Santee, CA, USA). Bone skeletal phosphatase was measured by an immunoradiometric method (Tandem-Ostase, Hybritech, USA).

C-Reactive Protein was determined by a standard immunonephelometric method (Behring, Scoppito, L’Aquila, Italy) with normal values <5 mg/L. ELISA by commercially available kits (R&D Systems Inc, Minneapolis, USA) was used to determine IL-6 (normal values < 3.13 pg/ml; coefficient of variation: intra-assay = 2.6%, inter-assay = 4.5%) and TNF- α (normal values <4.1 pg/ml; coefficient of variation: intra-assay = 4.7%, inter-assay = 5.8%) [16].

### Follow-up study

Fractures were defined as non-traumatic events documented by imaging techniques (i.e. radiography, computerized axial tomography or nuclear magnetic resonance). We considered fractures of the femoral neck (intertrochanteric, or subtrochanteric fractures), fractures of other parts of the femur (condyle, supracondylar, and epiphysis), vertebral fractures and fractures interesting other skeletal segments. Vertebral fractures were diagnosed using a semi-quantitative approach [17].

After enrollment patients were followed-up until the first fracture, those who died or underwent transplant or were free of fractures at the end of study were censored no patient was lost to follow-up. Median follow-up was 74 months (range 0.5-84.0 months).

### Statistical analysis

Data are reported as mean ± SD, median and inter-quartile range or as prevalence rate and differences between groups were analyzed by the *T*-test, the Mann–Whitney test or the Chi-Squared Test, as appropriate. The association between incident fractures with serum PTH, total and bone alkaline phosphatase, calcium, phosphate, TNF- α, CRP, Il-6 and others potential risk factors was preliminarily analyzed by dividing patients into two groups (patients with and without incident fractures) and by testing the differences between them. To identify patients with low and high bone turn-over we used intact PTH thresholds suggested by European Best Practice Group [18]. The predictive value of biomarkers of inflammation, mineral and bone disorder and other potential risk factors was analyzed by univariate Cox’s proportional hazards method. In the Cox’s analysis the proportional hazard assumption was tested by the analysis of Schoenfeld residuals and no violation was found. Due to the small number of fractures to assess the idependent link between TNF-α and fractures we entered this risk factor in(restricted) bivariate models considering other risk factors one at a time. Furthermore, we computed a risk score [19] in each patient by summing up the individual profile of 5 risk factors for fractures, each dichotomized as following : sex female = 1 male = 0, previous fractures = 1 no previous fractures = 0, previous transplants = 1 no previous transplants = 0, intact PTH > median value = 1 intact PTH < median value = 0, age > median value = 1 age < median value = 0. The potential confounding effect of this score, reflecting the combined effect of major risk factors for fractures, was then tested in an additional bivariate model.

Data are expressed as hazard ratio (HR) and 95% confidence interval (CI). All calculations were done using a standard statistical package (SPSS for Windows).

## Results

**Figure 1:**
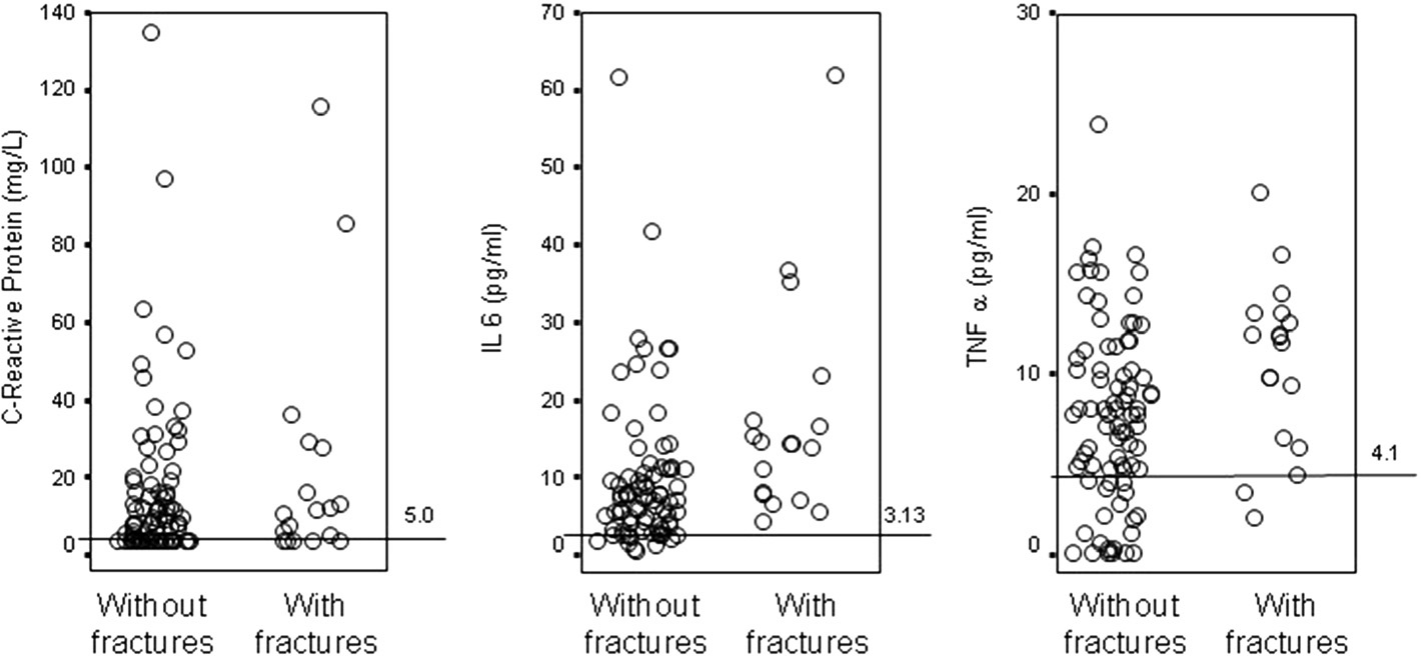
**Distribution of CRP, IL-6 and TNF-α levels.** Lines indicate the upper limit of the normal range.

**Figure 2:**
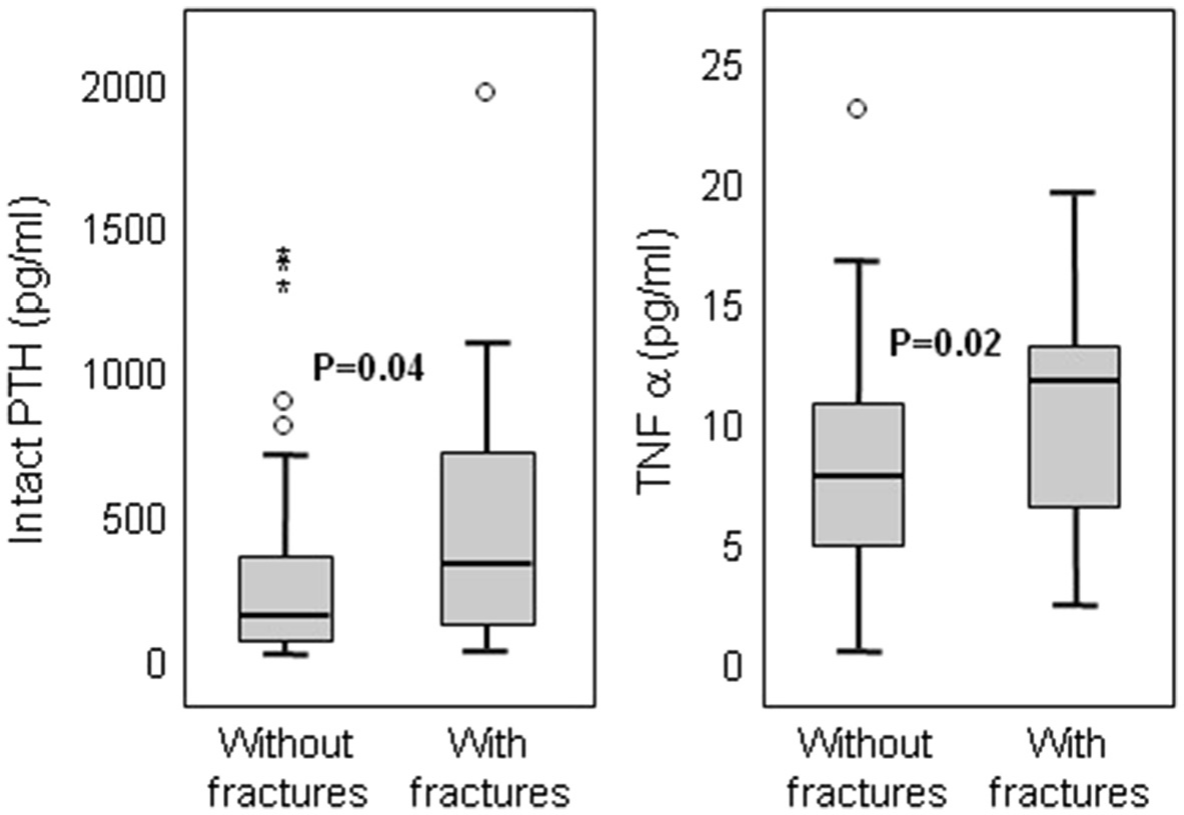
Serum intact PTH and TNF-α levels (median and interquartile range) in patients with and without incident fractures.

C-Reactive Protein, IL-6 a and TNF-α levels above the upper limit of the normal range were observed in 65%, 77% and 78% of patients, respectively (Figure 1). During the follow-up period (median 74 months; range: 0.5-84.0 months) 18 patients had incident fractures (vertebral = 10 ; pelvic = 4; femoral neck = 1; humerus = 1; costal = 1; clavicle = 1). Patients with incident fractures had higher levels of serum intact PTH, and TNF-α when compared to those without these complications (Table 1, Figure 2) while CRP and IL-6 levels were similar in the two groups. The proportion of patients with PTH in the range denoting low bone turn-over (<100 pg/ml) according to current guidelines [18] did not differ, while the proportion of those with high turn-over (>800 pg/ml) was higher among patients with incident fractures (Table 1). The proportion of patients who had suffered from a previous fracture was markedly higher in patients who had incident fractures (Table 1).

**Figure 3:**
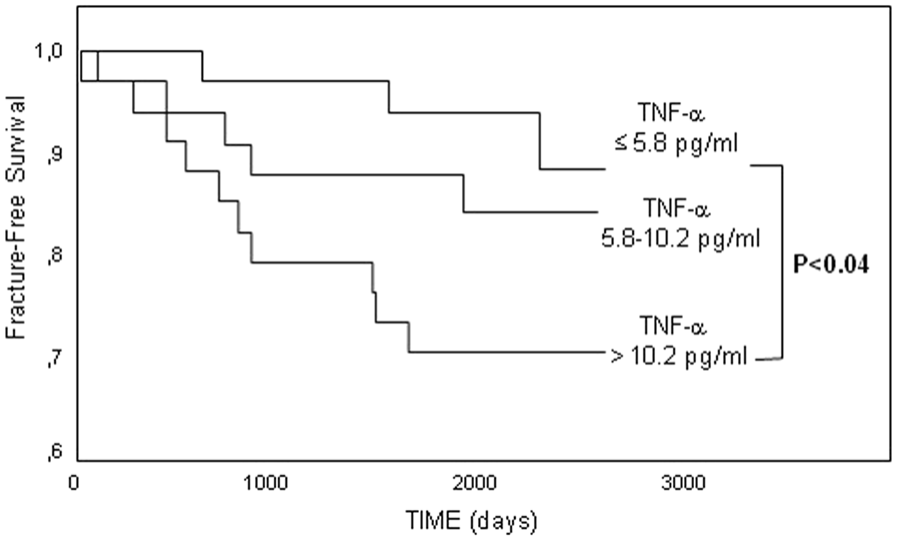
**Fracture-free survival according to TNF tertiles.** Kaplan-Meyer curves and log-rank test.

**Figure 4:**
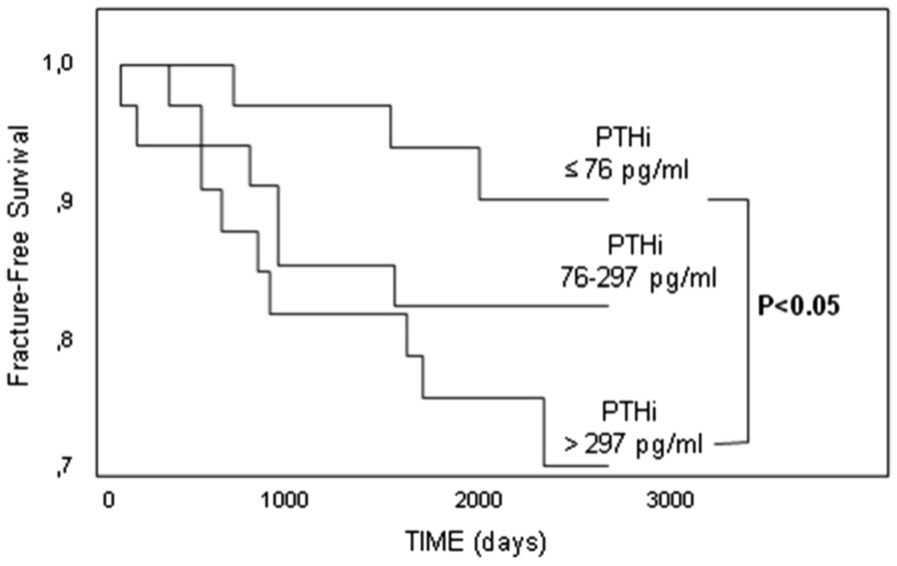
**Fracture-free survival according to PTH tertiles.** Kaplan-Meyer curves and log-rank test.

On Kaplan-Meyer analysis fracture-free survival was longer in patients with TNF-α in the lower tertile as compared to those with levels in the upper tertile (Figure 3). Similarly PTH levels in the lower tertile were associated to a longer fracture-free survival (Figure 4). On univariate Cox analysis the association between TNF-α with incident fractures was significant (HR for 5 pg/ml increase in TNF-α: 1.62 95% CI: 1.05-2.50; p = 0.03) as it was the association between fractures and PTH (HR for 100 pg/ml increase in PTH: 1.15 95% CI: 1.04-1.27; p = 0.005). History of previous fractures increased by four-fold the risk of incident fractures (HR 4.47 95% CI: 1.46-13.7; p = 0.009). CRP and IL-6, total and bone alkaline phosphatase, serum calcium and phosphate largely failed to predict fractures.

In reduced bivariate models the association of TNF-α with fractures proved to be independent of intact PTH, age, sex, history of previous transplants and previous fractures and of the risk score composed by the same risk factors (Table 2).

**Table 2:** Crude and adjusted relative risk of plasma TNF-α for incident fractures

	**Hazard ratio and 95% CI associated to 5 pg/mL increase in plasma TNF-α for incident fractures**
**Crude**	1.62 (1.05-2.50), P = 0.03
**Adjusted for:**	
Age	1.64 (1.06-2.56), P = 0.03
Intact PTH	1.55 (1.03-2.35), P = 0.04
Sex	1.64 (1.06-2.54), P = 0.03
Previous fractures	1.61 (1.01-2.57), P = 0.04
Previous transplants	1.59 (1.05-2.40), P = 0.03
Risk Score	1.60 (1.04-2.45), P = 0.03

Plasma TNF-α data were adjusted in bivariate Cox models including TNF-α and each risk factor listed. The latter factors were used to compute the risk score (see methods).

## Discussion

Our exploratory observations, confirming the central role of PTH, have shown that TNF-α is associated with incident fractures in CKD -5D population. These findings generate the hypothesis that systemic inflammation might contribute to increase the bone fracture risk in these patients.

In the last two decades the biology of osteoclast activation has been intensively investigated and the RANKL/RANK pathway emerged as a fundamental modulator of osteoclastogenesis [4]. Inflammatory cytokines are well established potent activators of the RANKL/RANK pathway [4-7] and play a direct role in osteoclastogenesis in post-menopausal women [20]. Furthermore, recent longitudinal studies [21-23] coherently suggest that high cytokines levels may contribute to bone loss and fractures in elderly women and men.

We once again confirm that inflammation is pervasive in CKD-5D patients [12-15]. Indeed, CRP levels were above the upper limit of the normal in as much as 65% of patients at time of enrolment. In the present study TNF-α was substantially increased, being above the upper normal range in 78% of patients, a figure close to that of other biomarkers of inflammation, but it was the only cytokine linked to the fracture risk. The finding is consistent with studies showing that among the cytokines, TNFα is the most powerful stimulator of osteoclastogenesis [4-7].

Our study has limitations. First, we measured TNF-α and other cytokines only once. Since the precision of the estimate of the usual level of inflammation biomarkers increases with repeated measures, the link between TNF-α and bone fractures might be even stronger than emerged in the present analysis. On the other hand, because of the low number of events, the possibility that the TNF-α − fractures link may merely represent a false positive finding cannot be dismissed. We controlled for confounding by adopting a parsimonious approach based on bivariate Cox models and a composite risk score [19], and found that the relationship between TNF- α and the risk of incident fractures proved to be independent of PTH, as well as of major confounders like age, sex, previous fractures and previous kidney transplants. Notwithstanding these adjustments failed to materially change the risk of fractures associated with high TNF-α, we cannot exclude residual confounding. A second obvious limitation is that we did not measure the full set of hormones controlling mineral balance including 25 hydroxy vitamin D and 1,25 hydroxy vitamin D and FGF23. Moreover we have not evaluated serum magnesium levels and did not study the relationships between inflammation and vascular calcifications. Finally, our data collected in a single Renal Unit cannot be generalized to the greater HD population. Due to these limitations our data are merely hypothesis generating. The relationship between fractures and inflammatory cytokines in CKD-5D patients needs to be confirmed in larger cohorts gathering a substantial higher number of bone events. The issue is of relevance because bone fractures in CKD-5D patients not only engender disabling orthopaedic problems but are also associated to increased mortality [24].

## Conclusions

In conclusion, our observations generate the hypothesis that TNF α plays a role in the increased risk of bone fractures in CKD-5D patients. Analyses in larger cohorts and with adequate number of events are needed to firmly establish the TNF α -fracture link emerged in the present study.

## Competing interests

No conflict of interest is related to this manuscript.

## Authors’ contribution

All Authors have made substantial contributions to conception and design, analysis and interpretation of data and have given final approval of the version to be published. RT and GT performed the statistical analysis. CZ and GE have been involved in drafting the manuscript. All authors read and approved the final manuscript.

## Pre-publication history

The pre-publication history for this paper can be accessed here:

http://www.biomedcentral.com/1471-2369/13/134/prepub
